# Effect of Processing on Reduction in Chiral Pesticide Hexaconazole for Kiwifruit Juice

**DOI:** 10.3390/molecules28166113

**Published:** 2023-08-17

**Authors:** Zelan Wang, Min Wang, Tianming Yang, Yao Wang, Dali Sun, Junxiao Pang

**Affiliations:** 1 The Key Laboratory of Environmental Pollution Monitoring and Disease Control, Ministry of Education, School of Public Health, Guizhou Medical University, Guiyang 550025, China; wzl2010kaoyan@163.com (Z.W.); wm1999929@163.com (M.W.); tianmingyang27@163.com (T.Y.); wygzykdx@163.com (Y.W.); 2 School of Food Science and Engineering, Guiyang University, Guiyang 550005, China

**Keywords:** kiwifruit, hexaconazole, risk assessment, residues, chiral pesticide, processing factors

## Abstract

In this study, the residue levels of chiral pesticide hexaconazole during kiwifruit juice processing (peeling, homogenization, and sterilization) were investigated by using high-performance liquid chromatography (HPLC), and the dietary risk during these processes was also assessed. Hexaconazole was applied at dosages of 173.33 and 346.66 mg/L (recommended and double recommended dosage) in kiwifruit. In the peeling process, 87.7% to 89.2% of the residues were decreased after peeling. Levels of hexaconazole residues in homogenization and sterilization processes further increased from 0.49% to 24.3% and from 0.2% to 3.0%, respectively. Processing factors (PFs) for (+)- and (−)-hexaconazole after peeling, homogenization, and sterilization were 0.12, 0.88, 0.99 for low-dose treatment and 0.12, 0.87, 0.99 for high-dose treatment, respectively. The enantioselectivity of hexaconazole during these procedures was evaluated by enantiomeric fractions (EFs) values, which were around 0.5 throughout all the procedures, indicating that hexaconazole enantiomers had similar dissipation behaviors during kiwifruit juice processing. The RQc of hexaconazole in pre-peeling samples was significantly greater than 100% under two dosages, while the peeling process can notably decrease the values to an acceptable level. The results of this study could provide guidance for agriculture applications and kiwi commodity production to decrease the risk of hexaconazole residue.

## 1. Introduction

Kiwifruit (*Actinidia chinensis*), also called “the king of fruits”, is described as a nutrient-dense fruit that contains abundant health-beneficial bioactive compounds like ascorbic acid, nutritional minerals, dietary fiber, carotenoids, polyphenols, etc. [1], which exert positive effects on cardiovascular disease [2], gut microbiota [3] and immune system [4]. Because of its nutritional and medicinal value, kiwifruit become one of the most commercialized fruits on the international market [5]. However, China, the place of origin of kiwifruit, is now the largest kiwifruit producer in the world with an annual output of approximately 1.06 million tons [6]. In addition to being consumed fresh, kiwifruit can be processed into numerous by-products such as juice, jam, vinegar, wine, and dried slices. However, kiwifruit can be infected with a variety of diseases such as phytophthora root, crown rot, botrytis fruit rot and bleeding canker caused by *phytophthora*, *botrytis cinerea* and *pseudomonas syringae pv. actinidiae*, respectively [7,8]. As a result, the application of fungicide is a necessity to control these kinds of diseases in kiwifruits.

**Figure 1:**
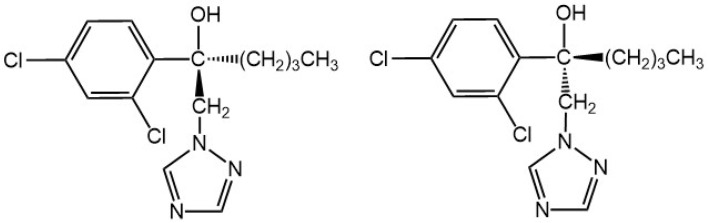
Chemical structures of hexaconazole enantiomers.

Hexaconazole, [(±)-α-butyl-α-(2,4-dichlorophenyl)-1,2,4,-triazole-1-ethanol], a member of triazole fungicides, exhibits excellent protective and curative capability against fungi diseases by interfering with ergosterol biosynthesis [9]. Since it is used as an agricultural fungicide, hexaconazole can also bio-accumulate in humans and wildlife or transport between ecosystems. It was reported that hexaconazole could interfere with the energy, amino acid and lipid metabolism of zebrafish and exhibit endocrine disruption activities [10]. Because of its toxicity, the United States Environmental Protection Agency (US EPA) has listed this compound as Group C—Possible Human Carcinogen [11]. It is worth noting that hexaconazole has an asymmetrically substituted carbon atom and contains a pair of enantiomers (Figure 1), which exhibits differences in environmental behavior, agricultural product processing, toxicities, or biological macromolecule-mediated metabolisms. For instance, (−)-hexaconazole achieves more antifungal activity than (+)-hexaconazole, while (+)-hexaconazole degrades faster than its antipode in tomato [12,13]. Thus, paying attention to these two enantiomers might be a more realistic reflection of their toxicity and risk in food.

Food safety has drawn worldwide concern due to various pollution sources, especially the extensive application of pesticides in agricultural production. Studies showed that nearly 42% of vegetable samples from the North-Western Himalayan region of India exceeded the maximum permissible limits (MRL) established by European Commission, and hexaconazole was one of the most frequently detected pesticides in these samples [14]. Another study conducted in the Incheon area of Korea showed that residues were detected in 9.5% of vegetables, and 1.7% of them had exceeded the Korean MRLs. Similarly, the most frequently detected was hexaconazole [15]. In addition, residues and dissipation of hexaconazole in mango, curry leaves and black tea have been reported recently [16,17,18]. However, terminal residue determination and dissipation behavior estimation of hexaconazole in kiwifruit are urgently needed. For food safety and environmental protection, several government agencies have established the MRL of hexaconazole in kiwifruit. According to the EU pesticides database of the European Commission, the MRL of hexaconazole was set at 0.01 mg/kg [19]. In Japan, the Food Chemical Research Foundation has set the limit of hexaconazole at 0.2 mg/kg in kiwifruit [20], while in China, the value was set at 3 mg/kg according to the national standard [21]. The MRL for hexaconazole in kiwifruit can provide strong support for the effective management of pesticide residues in fruit by local regulatory authorities and also can provide reliable data for the revision of the MRLs in the future. Hence, research regarding residue determination and dissipation behavior assessment of hexaconazole in kiwifruit is all equally useful.

In addition to the application concentrations, the residue levels of pesticides in harvested agricultural products can be influenced by several factors such as their physicochemical properties, processing, and storage conditions. Studies have shown that household as well as industrial food processing steps including washing, peeling, boiling, steaming, blanching, and juicing could decrease pesticide residues in food [22]. It was reported that volatilization, hydrolysis, and thermal degradation caused a reduction in pesticide residues in the range of 20.7–100% [23]. However, the processing factors (PFs) of dimethomorph, famoxadone and cymoxanil in grapes drying were 1.03, 1.95 and 1.99, respectively, which indicates that the process of drying could concentrate the residues [24]. The production of kiwifruit juice typically includes sorting and washing, pressing, filtering, blending, homogenization, sterilization, and canning [25]. However, the effects of the above processing methods on hexaconazole residues in kiwifruit are still unclear. Moreover, the different dissipation behaviors of these two enantiomers during the processes are not intensively worked upon to the best of our knowledge.

Therefore, to clarify the effects of kiwifruit processing on residue levels and the enantioselective characters of this chiral pesticide hexaconazole, kiwifruits were treated with hexaconazole SC formulation, and common industrial processing methods were applied. Finally, samples at each processing step were detected and the pesticide residues were determined by using HPLC. The results of this research give insights into the methods of pesticide removal during the processing of kiwifruit juice.

## 2. Results

### 2.1. Method Validation

**Figure 2:**
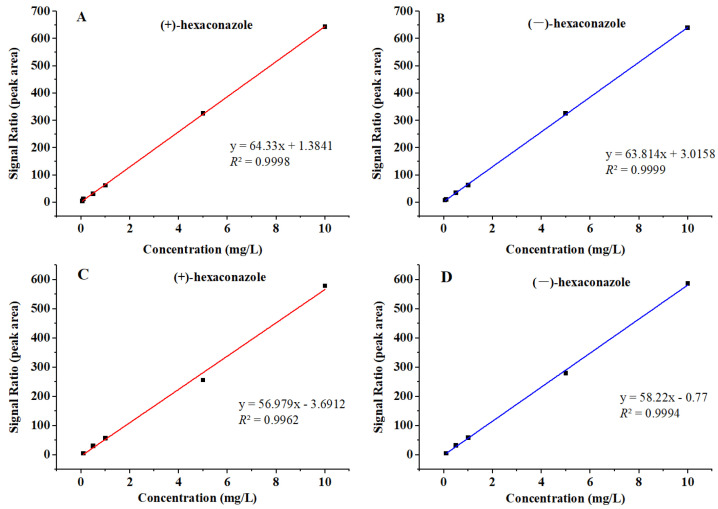
Calibration curves of (+)-hexaconazole and (−)-hexaconazole in hexaconazole solvent (**A**,**B**) and matrix-matched standards (**C**,**D**).

The calibration curves for hexaconazole enantiomers were established by plotting the peak areas (y) against the analyte concentrations (x). Good linearities were achieved with linear equations where y = 64.33x + 1.384 and y = 63.81x + 3.016 in hexaconazole solvent, y = 56.98x − 3.691 and y = 58.22x − 0.770 in matrix-matched standards. The correlation coefficients (*R*^2^) were both above 0.99 for (+)- and (−)-hexaconazole (Figure 2). The LOD and LOQ were estimated as signal-to-noise (S/N) ratios of 3 and 10, which were 0.01 and 0.05 mg/kg, respectively, for hexaconazole in kiwifruit. The accuracy and precision of this established method were evaluated by recovery rate with five replications (*n* = 5) at three fortified levels (0.05, 2 and 5 mg/kg). The recoveries for (+)- and (−)-hexaconazole enantiomers ranged from 91.55 to 96.86% and from 93.84 to 97.32% with relative standard deviations (RSDs) ranging from 1.86 to 8.75% and 1.95 to 9.40%, respectively (Table 1). The stability of the instrument was also assessed by determining the RSDs of inter-day and 3-day continuous injection of the same sample, and the results showed that the RSDs of inter-day injection were 0.56% and RSDs of 3-day injection were 2.43%. The results of method validation met the requirements of China’s national standard [26] and also satisfied the requirements of determination of hexaconazole enantiomers in kiwifruit and its processing products. 

### 2.2. Enantiomeric Separation by HPLC

**Figure 3:**
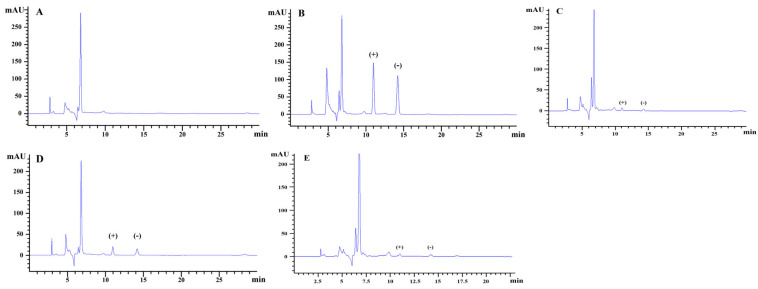
Typical HPLC chromatograms of hexaconazole enantiomers: (**A**) kiwifruit blank sample, (**B**) hexaconazole matrix-matched standards (5 mg/L), (**C**) peeled kiwifruit samples, (**D**) homogenized kiwifruit samples, (**E**) sterilized kiwifruit samples at recommended dosage.

The HPLC chromatograms of hexaconazole enantiomers were shown in Figure 3. According to the established detection conditions, the enantiomers of hexaconazole were separated completely with retention times of 10.98 min for (+)-hexaconazole and 14.10 min for (−)-hexaconazole, respectively.

### 2.3. Effects of Kiwifruit Juice Processing on Residues of Hexaconazole

**Figure 4:**
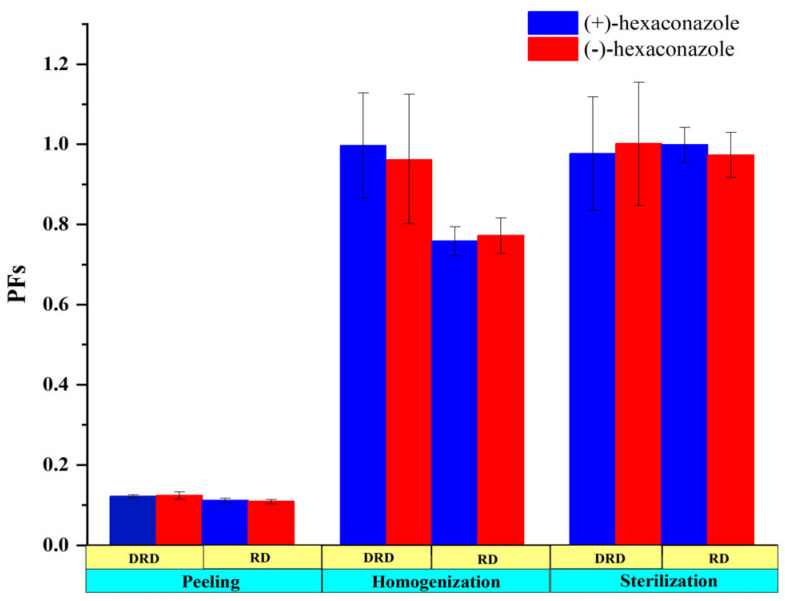
Processing factors (PFs) of (+)- and (−)-hexaconazole during kiwifruit juice processing. DRD and RD refer to double recommended and recommended dosages, respectively.

The residues of hexaconazole during each processing step of kiwifruit juice were shown in Table 2 and Table 3. According to the results, when the pesticide was applied at a dosage of 173.33 mg/kg, concentrations of (+)- and (−)-hexaconazole in the kiwifruit sample were 14.45 and 13.97 mg/kg, respectively. Nevertheless, when the kiwifruit was peeled, the residue levels reduced to 1.60 and 1.51 mg/kg, respectively. Similarly, when the pesticide was applied at a dosage of 346.66 mg/kg, residues of (+)- and (−)-hexaconazole in the kiwifruit sample were 33.43 and 32.87 mg/kg and both decreased to 4.06 mg/kg after peeling. The dissipation rate of hexaconazole residues in homogenization and sterilization processes further increased from 0.49% to 24.3% and from 0.2% to 3.0%, respectively, at two application dosages. The PF values were further used to clarify the effects of processing on the residue levels of hexaconazole enantiomers (Figure 4). The PFs of the peeling process for (+)- and (−)-hexaconazole at recommended and double recommended dosages were both 0.11 and 0.12, suggesting that the residues of hexaconazole enantiomers in kiwifruit can be effectively decreased by removing the peel. These results were in line with Roccio’s research that suggested that the low penetration ability of pesticide from peel into pulp is most likely due to the impermeable structure of its peel [27]. Moreover, the octanol–water partition coefficient (log_Kow_) and the solubility of pesticides might also influence their adsorption ability. These results were further verified by Kid et al., who reported that hexaconazole had an octanol–water partition coefficient of 3.9 at 20 °C and was registered as an intermediate adsorption pesticide [28].

**Figure 5:**
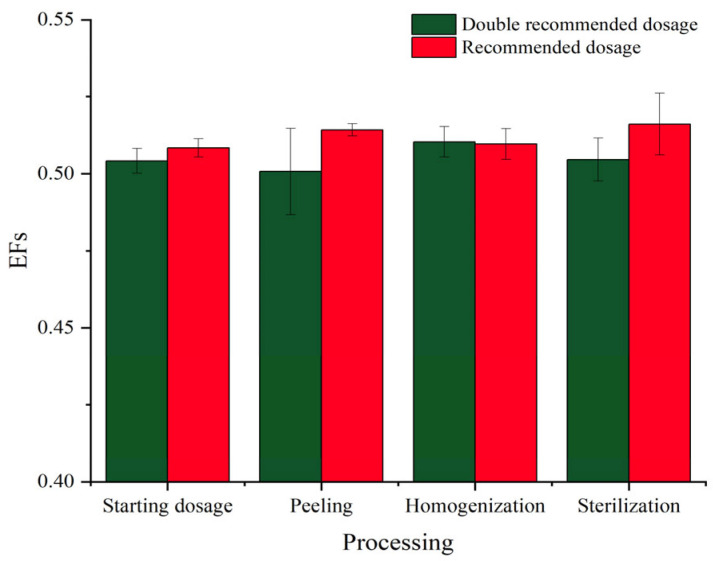
EFs of hexaconazole enantiomers at recommended and double recommended dosages during kiwifruit juice processing.

The enantioselectivity of hexaconazole during processing steps can be evaluated by EF values. As shown in Figure 5, the EFs of hexaconazole enantiomers after soaking, peeling, homogenization, and sterilization were 0.508, 0.514, 0.510, and 0.516 for the recommended dosage and 0.504, 0.500, 0.510, and 0.505 for double the recommended dosage, respectively. EFs of each processing were around 0.5, indicating similar degradation behavior of hexaconazole enantiomers during kiwifruit juice processing. These results were similar to a previous report, which suggested that there was no enantioselectivity of tetraconazole in the initial strawberry samples before fermentation in the process of wine-making [29]. However, the existence of yeast and microorganisms in strawberry juice contributed to the enantioselective process of the chiral compound. In addition, enzyme systems were supposed to play an important role in the enantioselective metabolism of many chiral pesticides, but that was on the condition of sufficient time for metabolism [30]. In the present study, however, healthy kiwifruits were selected with a small number of microorganisms and there were no extraneous ones introduced into the kiwifruit juice, together with the short exposure duration, so little enantioselectivity was observed during those procedures.

### 2.4. Dietary Risk Assessment of Hexaconazole during Kiwifruit Juice Processing

According to the China Statistical Yearbook, the average daily consumption of kiwifruit is 41.8 g/d, and the maximum consumption is 52.9 g/d. The average adult weight in China is 63 kg [31]. The acceptable daily intake of hexaconazole is set as 0.005 mg/kg bw·d [21]. According to the formulas described above, the RQc of hexaconazole residues in kiwifruit could be calculated. However, due to the absence of ARfD, the RQa of hexaconazole cannot be evaluated.

The MRL of hexaconazole in kiwifruit was set at 3 mg/kg [21]. Initial concentrations of hexaconazole enantiomers in kiwifruit before peeling were 4.7 and 11.1 times the MRL value in the recommended and double the recommended dosages, respectively. However, after peeling, residues in the recommended dose group were significantly reduced and were below the MRL., while for the double recommended dose group, hexaconazole residues still exceeded the Chinese standard after peeling, which implied the potential health risks via food consumption. Moreover, the application dosage could directly affect the safety of agricultural products which we should pay more attention to. Thus, dietary risk assessments were conducted to evaluate the edible risk degree of hexaconazole in kiwifruit.

Dietary exposure assessment of hexaconazole residues in kiwifruit juice processing showed that the NEDI values for both enantiomers calculated according to Equation (3) were between 0.0008–0.0096 and 0.0026–0.0222 mg/(kg·bw·d), respectively, for recommended and doubled recommended dosages (Table 2 and Table 3). Moreover, the NEDI at initial concentrations of two dosages significantly exceeded the ADI of hexaconazole (0.005 mg/kg bw·d). Correspondingly, RQcs of (+)- and (−)-hexaconazole before peeling were 191.69%, 185.33% for the recommended dosage, and 443.58%, 436.20% for double the recommended dosage, respectively. However, these values reduced to 21.27%, 20.08%, and 53.88%, 53.81% after peeling, which indicated that the chronic dietary risk of hexaconazole residues in kiwifruit juice before peeling was unacceptable for humans, while the risk decreased by almost 9-fold after peeling. The high RQc values of these samples before peeling might be attributed to the following: (1) Long immersion time and shorter harvest interval. In the present study, field application was simulated by pesticide soaking, and the soaking process may result in higher levels of hexaconazole. However, there was no harvest interval in this study like field experiment. (2) Structure of kiwifruits. Kiwifruits used in the present study had been already ripped when the simulated pesticides were applied; thus, a large amount of fluff on the surface of fruits would lead to more pesticide residues. (3) Different environmental conditions. Under field conditions, natural environmental factors such as rainfall, sunlight, and temperature are critical factors influencing hexaconazole dissipation, degradation, and persistence character [32]. For instance, high temperatures could increase the dissipation rates of carbendazim, thiabendazole and procymidone in Shiitake [33]. However, these conditions cannot be achieved due to the in-house experiment in this study. 

## 3. Materials and Methods

### 3.1. Chemicals and Instruments

Hexaconazole standard dissolved in acetone (100 mg/L) was purchased from Dr. Ehrenstorfer GmbH (Augsburg, Germany). Hexaconazole suspension concentrate formulation (40% active ingredient) was supplied by Limin Agrochemicals Co., Ltd. (Xuzhou, China). Acetonitrile and methanol with HPLC grade were obtained from CNW Technologies GmbH (Düsseldorf, Germany). Anhydrous magnesium sulfate (MgSO_4_), acetic acid, primary secondary amine (PSA), sodium chloride (NaCl), octadecylsilyl (C_18_), potassium permanganate (KMnO_4_), sugar and citric acid were of analytical grade and purchased from Chuandong Chemical Co., Ltd. (Chongqing, China). High-performance liquid chromatography (HPLC) analyses were conducted with Agilent 1260 HPLC system (Agilent Technologies, Inc., Santa Clara, CA, USA). Chromatographic separation of hexaconazole enantiomers was performed using Lux^®^ Cellulose-2 chiral column (250 mm × 4.6 μm, 5 μm particle size) (Phenomenex Inc. Torrance, CA, USA). Other instruments included a thermostatic water bath (HH-W600; Olebo, Jinan, China), a squeezer (JS39D-250; Supor, Hangzhou, China), a sterilizer (BXM-30R; Boxun, Shanghai, China), a homogenizer (FJ200-SH; Huxi, Shanghai, China) and a Milli-Q system (Bedford, MA, USA). 

### 3.2. Sampling of Kiwifruit

Kiwifruits were purchased from a local market in Guiyang, China. Healthy and mature ones were selected and rinsed with clean water 2–3 times after the treatment of 0.1 mg/L potassium permanganate solution. After that, kiwifruits were soaked with 173.33 or 346.66 mg/L hexaconazole solutions prepared with 1.3 or 2.6 mg hexaconazole SC formulation diluted with 3 L water. The control group was carried out with the same amount of water. After soaking for 4 h, kiwifruit samples were taken out, dried under natural air, and moved to the processing steps.

### 3.3. Kiwifruit Juice Processing

In general, kiwifruit juice processing includes three steps. Process 1 (P1): peeling and juicing. Kiwifruit samples were peeled, crushed, filtered, and centrifuged at 10,000 r/min for 10 min, and 5 g of supernatant was adopted for the quantitative determination of hexaconazole enantiomers. Process 2 (P2): blending and homogenization. Fifty grams of supernatant samples were weighed exactly and mixed with sugar, citric acid, stabilizer, and essence, which were added at 40% of the weight of the original juice so that the soluble solid content was above 35%. Then, the mixture was homogenized and the concentrations of hexaconazole were determined. Process 3 (P3): sterilization and canning. After sterilization at 120 °C for 15 min, samples were quickly canned at a temperature of 70~80 °C and a vacuum degree of over 46.7 kPa. Finally, the juice was made and placed at 30 °C. 

### 3.4. Extraction and Cleanup 

For hexaconazole determination, 5 g of kiwifruit juice from each processing step was weighed into a 20 mL Teflon centrifuge tube and 7.5 mL of acetonitrile was added. Samples were conducted with ultrasonic extraction for 15 min. After that, 4 g anhydrous MgSO_4_ and 1 g NaCl were added, vortexed vigorously and centrifuged at 4000 r/min for 5 min. One milliliter of the upper layer (acetonitrile) was transferred into a new 2 mL centrifuge tube containing 50 mg PSA, 50 mg C18 and 150 mg anhydrous MgSO_4_. The mixture was thoroughly vortexed for 1 min again and subsequently centrifuged at 4000 r/min for 5 min. The upper layer was transferred to an auto-sampler vial for HPLC analysis.

### 3.5. HPLC Detection

An Agilent 1260 series HPLC and Lux^®^ Cellulose-2 chiral column (250 mm × 4.6 μm, 5 μm particle size) were used for the separation and detection of hexaconazole enantiomers with an injection volume of 20 μL. Acetonitrile and ultrapure water were used as mobile phases with a ratio of 70/30 (*v*/*v*) and a flow rate of 0.3 mL/min. The column temperature was kept at 30 °C and the UV wavelength was set at 210 nm. According to a published study, the first eluted enantiomer was confirmed as (+)-hexaconazole and the second one was (−)-hexaconazole on the chiral column [34].

### 3.6. Method Validation

Method validation in this study was carried out according to the agricultural industry standards of China and was composed of calibration curve linearity, limit of detection (LOD), limit of quantification (LOQ), accuracy and repeatability [26]. The working standard solutions of hexaconazole (0.05, 0.1, 0.5, 1.0, 5.0 and 10 mg/L) were serially diluted from the 100 mg/L stock standard solution using acetonitrile, by which the calibration curves of hexaconazole enantiomers were drawn. Meanwhile, calibration curves of (+)-hexaconazole and (−)-hexaconazole in the kiwifruit juice matrix were obtained by adding the above hexaconazole standard solutions to the kiwifruit blank samples. Data on pesticide residues and risk assessment in this research were all calculated based on the calibration curves of matrix-matched standards. Fortified recoveries were carried out to investigate the accuracy and precision of this established method by adding hexaconazole standard solutions to the blank kiwifruit samples at the concentrations of 0.05, 2 and 5 mg/kg, respectively. The limit of detection (LOD) was defined as the lowest concentration of samples when the signal-to-noise (S/N) ratio was 3, and the limit of quantification (LOQ) was determined as an S/N ratio of 10.

### 3.7. Enantiomeric Fraction (EF)

The EF value of hexaconazole was calculated to measure the enantioselectivity of enantiomers during the kiwifruit juice processing steps. EF is defined as below:

EF = Concentration of (+)-hexaconazole/[(+)-hexaconazole + (−)-hexaconazole](1)

The EF values ranged between 0 and 1. The value of 0.5 indicates a racemic mixture, while EF > 0.5 or EF < 0.5 indicates preferential degradation of the (−)- or (+)-enantiomer, respectively [35].

### 3.8. Determination of Processing Factor (PF)

The effect of household or industrial processing on pesticide residues in food often correlates with the physicochemical properties of the pesticides. To investigate the effects of processing methods on hexaconazole residues, the PF values for all processing steps were calculated by the ratio of the pesticide residues in the processed commodity (mg/kg) to the pesticide levels in the raw commodity (mg/kg) [36]:

PF = Residue levels of processed commodities/the raw commodities(2)

According to the equation, PF < 1 indicates a reduction in pesticide concentration during processing, while PF > 1 is known as a concentration accumulation.

### 3.9. Risk Assessment

The risk assessment of dietary exposure to pesticide residues in food is based on the amount of pesticide intake and is calculated according to the following formulas [37]:

NEDI = STMR × F/bw(3)

RQc = NEDI/ADI(4)

NESTI = HP × LP/bw(5)

RQa = NESTI/ARfD(6)

where NEDI represents the national estimated daily intake, ADI is the acceptable daily intake, NESTI refers to the national estimated short-term intake and ARfD is the acute reference dose. All are expressed as μg/(kg bw·d). STMR refers to supervised trials’ median residue (mg/kg), F is consumption of foods (g/d), bw is body weight (kg), HR is highest residue (mg/kg), LP represents the largest consumed portion (g/d), and RQc and RQa refer to chronic and acute risk quotient, respectively. The dietary risk of pesticides for humans is considered unacceptable when RQc or RQa is higher than 100% and a higher value indicates a greater risk, while RQc or RQa less than 100% carries the opposite meaning.

### 3.10. Data Analysis

All the treatments and procedures including peeling, homogenization, and sterilization were conducted in three replicates. Hexaconazole enantiomer residues in kiwifruit after each processing method were expressed as average values ± RSDs. Because of the non-normal distribution data, the statistical differences were checked using the non-parametric Kruskal–Wallis test with SPSS v20.0 software (SPSS, Inc., Chicago, IL, USA). The difference at a *p* value < 0.05 was considered statistically significant in this study. Origin Pro v9.8 (OriginLab, Northampton, MA, USA) was used to draw statistical graphs.

## 4. Conclusions

In the present study, a simple, sensitive, and effective HPLC method was developed to determine the triazole fungicide hexaconazole enantiomers in kiwifruit juice processing. Their enantioselectivity behaviors during different kiwifruit juice procedures (peeling, homogenizing, and sterilizing) were explored and the dietary risk of hexaconazole in kiwifruit juice at two dosages were also evaluated. According to the results, the established method showed good linearity, accuracy, and precision to determine hexaconazole enantiomers. The procedure of peeling could effectively reduce residues of (+)- and (−)-hexaconazole both in recommended and double recommended dosages with low PF values during kiwifruit juice processing. In addition, hexaconazole enantiomers exhibited similar degradation behavior during kiwifruit juice processing with EFs fluctuating around 0.5. Dietary exposure assessment showed that the chronic dietary risk of hexaconazole residues in kiwifruit juice before peeling is unacceptable, but was acceptable in peeled samples. These findings might be useful for the enantioselectivity investigation of the chiral pesticide in food and provide guidelines for kiwifruit juice production.

## Figures and Tables

**Table 1:** Recoveries and RSDs of hexaconazole enantiomers at three fortified levels.

Enantiomers	Fortified Levels (mg/kg)	Recoveries(% ± SD) (*n* = 5)
(+)-hexaconazole	0.05	96.57 ± 8.75
	2	91.55 ± 2.19
	5	96.86 ± 1.86
(−)-hexaconazole	0.05	94.90 ± 9.40
	2	93.84 ± 3.58
	5	97.32 ± 1.95

**Table 2:** Residues of hexaconazole enantiomers during kiwifruit juice processing and the dietary exposure risk at recommended dosage.

Processing	Enantiomer	Residue (mg/kg)	STMR (mg/kg)	HR (mg/kg)	NEDI mg/(kg bw·d)	NESTI mg/(kg bw·d)	RQc (%)	RQa (%)
Starting dosage	(+)	14.17–14.68	14.45	14.68	0.0096	0.0123	191.69	-
	(−)	13.87–14.10	13.97	14.10	0.0093	0.0118	185.33	-
Peeling	(+)	1.55–1.70	1.60	1.70	0.0011	0.0014	21.27	-
	(−)	1.45–1.60	1.51	1.60	0.0010	0.0013	20.08	-
Homogenization	(+)	1.20–1.22	1.21	1.22	0.0008	0.0010	16.09	-
	(−)	1.15–1.17	1.17	1.17	0.0008	0.0010	15.48	-
Sterilization	(+)	1.17–1.25	1.21	1.25	0.0008	0.0010	16.06	-
	(−)	1.08–1.19	1.13	1.19	0.0008	0.0010	15.06	-

**Table 3:** Residues of hexaconazole enantiomers during kiwifruit juice processing and the dietary exposure risk at double recommended dosage.

Processing	Enantiomer	Residue (mg/kg)	STMR (mg/kg)	HR (mg/kg)	NEDI mg/(kg bw·d)	NESTI mg/(kg bw·d)	RQc (%)	RQa (%)
Starting dosage	(+)	33.29–33.71	33.43	33.71	0.0222	0.0283	443.58	-
	(−)	32.41–33.73	32.87	33.73	0.0218	0.0283	436.20	-
Peeling	(+)	3.91–4.19	4.06	4.19	0.0027	0.0035	53.88	-
	(−)	3.65–4.32	4.06	4.32	0.0027	0.0036	53.81	-
Homogenization	(+)	3.52–4.40	4.04	4.40	0.0027	0.0037	53.65	-
	(−)	3.45–4.17	3.87	4.17	0.0026	0.0035	51.38	-
Sterilization	(+)	3.50–4.33	3.92	4.33	0.0026	0.0036	52.02	-
	(−)	3.38–4.19	3.85	4.19	0.0026	0.0035	51.08	-

## Data Availability

Data are contained in the manuscript.

## Sample Availability

Samples of the compounds Hexaconazole enantiomers are available from the authors.
